# Beyond Overdiagnosis: Evaluating the Drivers of Increasing Thyroid Cancer Incidence in the Era of Obesity, Environmental Exposure, and Advanced Diagnostics

**DOI:** 10.3390/cancers18142193

**Published:** 2026-07-08

**Authors:** Marta Druszcz, Justyna Łapińska, Igor Kusio, Wiktoria Krowisz, Klaudia Gładowska, Weronika Pająk, Jakub Kleinrok, Ryszard Sitarz, Agnieszka Korolczuk

**Affiliations:** 1 Department of Clinical Pathomorphology, Medical University of Lublin, Jaczewskiego 8b, 20-090 Lublin, Poland; marta.druszcz@interia.pl (M.D.); lapinskaju@gmail.com (J.Ł.); kusza03@wp.pl (I.K.); wiktoriakrowisz@gmail.com (W.K.); agnieszka.korolczuk@umlub.edu.pl (A.K.); 2 Student Scientific Society of Forensic Medicine, Department of Correct, Clinical and Imaging Anatomy, Medical University of Lublin, 20-810 Lublin, Poland; kmgladowska@gmail.com; 3 1st Department of Psychiatry, Psychotherapy and Early Intervention, Medical University of Lublin, Gluska Street 1, 20-439 Lublin, Poland; ryszard.sitarz@umlub.edu.pl

**Keywords:** thyroid cancer, epidemiology, incidence trends, overdiagnosis, obesity, endocrine-disrupting chemicals, environmental exposures, molecular drivers

## Abstract

The global incidence of thyroid cancer (TC) has increased significantly in recent decades. Traditionally, this trend has been attributed to overdiagnosis resulting from the widespread use of imaging and improved diagnostic techniques, but increasing evidence suggests that current epidemiological patterns may not be fully explained by diagnostic factors alone. Most importantly, increasing rates of larger and more advanced tumors have been reported in several populations. This narrative review discusses the evidence on possible drivers of rising TC incidence: obesity and metabolic dysfunction, environmental exposures such as air pollution, and endocrine-disrupting chemicals (EDCs) and ionizing radiation, as well as molecular and genetic mechanisms involved in thyroid carcinogenesis. The role of advances in diagnostics and their contribution to overdiagnosis are also discussed. Current available data support a multifactorial model with better detection coupled with changes in environmental, metabolic and biological risk factors. A better understanding of the relative contribution and interaction of these determinants may help refine diagnostic strategies, reduce unnecessary interventions, and improve the prevention and management of TC.

## Simple Summary

Thyroid cancer is being diagnosed more often than ever before. The reasons behind this increase were previously attributed to improved medical imaging and to the finding of small tumors which would have been missed earlier. Recent observations indicate that this explanation may not be completely suitable for current trends. This review aims to explore the variety of factors that have been considered as possible contributors to the increased occurrence of thyroid cancer, including obesity, metabolic disorders, environmental pollution, and radiation exposure, as well as changes in biology of the tumors. It also discusses the role of advancements in diagnosis on detection of the disease.

## 1. Introduction

The most common malignant neoplasm of the endocrine system is thyroid cancer (TC), which according to the 2022 data of the International Agency for Research on Cancer (IARC) GLOBOCAN, ranked 7th in terms of incidence worldwide. It constitutes 4.1% of all cancer diagnoses, with a very high five-year survival rate—reaching as much as 98.5% [1,2]. Since 1980, a global upward trend in TC has been observed—which persisted until 2010—among others in the USA and Republic of Korea. Following that, TC incidence declined significantly in certain populations, while the number of deaths remained relatively stable [2,3]. The risk of developing the disease increases with age and the global index clearly indicates that TC occurs three times more often in women than in men. Significant risk factors include exposure to radiation, iodine intake, as well as obesity, metabolic syndrome, family predisposition, and genetic and molecular factors [1,2]. Thyroid cancer arises mainly due to somatic genetic alterations, and its nature is sporadic, accounting for as much as 90% of all thyroid cancers; the remaining cases are familial forms, which include medullary thyroid cancer and non-medullary thyroid cancer. Familial non-medullary thyroid cancer is generally defined by the occurrence of the disease in more than two first-degree relatives, because family history in this case is considered a marker of genetic risk [4,5]. Globally widespread examinations—including thyroid ultrasound, fine-needle aspiration biopsy (FNAB), and various imaging techniques—caused an intensive increase in diagnosed cases of TC, leading to overdiagnosis, which particularly affected low- and middle-income countries (LMICs) and countries with a high population density such as China or Brazil [1,2,3]. This is clearly demonstrated by the Cancer Incidence in the Five Continents database from 2008 to 2012, which showed that overdiagnosis accounted for 34–93% of TC diagnoses, as well as by the IARC Global Cancer Observatory database from 2013 to 2017, which estimated 1.7 million excess TC diagnoses attributable to overdiagnosis across 63 countries [2,3]. Although overdiagnosis remains the main factor in the increasing incidence of TC, the observed trend also includes an increase in the number of cases in advanced stages of the disease. One of these is papillary thyroid carcinoma (PTC), for which the Surveillance, Epidemiology, and End Results (SEER) cancer registry in the USA demonstrated a slower but increasing incidence trend amounting to 3.5% since 1981, as well as an increase in mortality among individuals with advanced-stage cancer amounting to 2.9% annually in the years 1994–2013. In Queensland, Australia, the incidence rate of differentiated thyroid carcinoma (DTC) stage III/IV significantly increased relative to age from 0.54 (1982–1986) to 1.18 (2002–2008) [1]. Contemporary studies indicate that the increase in TC incidence is a multifactorial phenomenon in which, apart from overdiagnosis, environmental factors are also involved, including ionizing radiation used as a result of treatment and resulting from environmental contamination caused by nuclear accidents [6]. Environmental chemicals or mixtures of chemicals that disrupt hormone function, referred to as endocrine-disrupting chemicals (EDCs), have attracted significant attention. Comprehensive analyses have demonstrated associations between exposure to several EDCs and an increased risk of thyroid cancer, although further studies are needed to fully understand the underlying biological mechanisms [7]. Metals are naturally occurring environmental elements, some of which are essential for life, whereas others may exhibit toxic and carcinogenic properties even at low concentrations. Environmental exposure to metals may result from both natural and anthropogenic sources, and studies have suggested a positive association between metal contamination and thyroid cancer incidence [8]. Polluted air is a mixture of countless substances, including particulate matter with an aerodynamic diameter of ≤2.5 μm. In 2013, the International Agency for Research on Cancer, as well as data from the Korean National Health Insurance Service from 2002 to 2015—where the association between thyroid cancer and exposure to meteorological conditions and air pollution over a period of 3 years was investigated—showed that meteorological parameters, as well as air pollution—including increasing PM2.5 concentrations—were significantly associated with thyroid cancer [9,10]. The association between pesticide exposure and thyroid cancer is currently the subject of intensive epidemiological research, although available findings remain inconsistent due to the limited number of studies and differences in exposure assessment methods [11]. Obesity, being the second most common modifiable factor increasing the development of TC, is supported by an extensive meta-analysis of the impact of obesity indicators on TC risk amounting to 1.16 with a corresponding 95% confidence interval (CI) [12]. Another factor is age, particularly above 55 years, among whom the global incidence increased by 185% in the years 1990–2021 [13]. The increase in the number of cases in more advanced stages of the disease and the growing importance of environmental and metabolic factors suggest that factors beyond overdiagnosis contribute to this trend. Therefore, a comprehensive reassessment of the factors responsible for the increasing incidence of TC is necessary, taking into account both the influence of overdiagnosis and actual changes in disease occurrence.

The aim of this study is the evaluation of the multifactorial causes of the increase in TC incidence, the explanation of which is not based exclusively on overdiagnosis. The study aims to present current epidemiological, clinical, molecular, environmental, metabolic, and diagnostic data in order to better demonstrate incidence trends, assess the limitations of overdiagnosis as the sole explanation, and analyze the role of obesity, metabolic disorders, environmental exposure, genetic changes, and advances in diagnostic technologies.

## 2. Materials and Methods

### 2.1. Study Design

This study is a narrative review aiming to collect current knowledge on increased incidence rates of TC, particularly regarding the role of the relative contributions of overdiagnosis, metabolism, environment, and molecular mechanisms.

Considering the complexity of the topic, a narrative approach was selected over a systematic review to incorporate different types of data such as epidemiological trends, mechanistic insights, and clinical observations. This methodology allows for developing hypotheses concerning causal pathways and interactions between these factors. To improve comprehensiveness of the study, elements of systematic literature searching were adapted to inform this narrative synthesis. However, the review does not aim to provide quantitative evidence synthesis.

### 2.2. Literature Search Strategy

A structured literature search was conducted in PubMed/MEDLINE, Scopus, and Web of Science for studies published up to May 2026. Search terms combined keywords and Medical Subject Headings (MeSH) related to TC incidence, overdiagnosis, obesity, metabolic dysfunction, environmental exposures, molecular mechanisms, and diagnostic technologies.

Additional targeted searches were conducted for specific environmental risk factors, including air pollution, EDCs, heavy metals, and ionizing radiation. Reference lists of relevant articles were also screened to identify additional studies.

### 2.3. Eligibility Criteria and Study Selection

Eligible publications included original research articles, systematic reviews, and meta-analyses addressing TC incidence trends, risk factors, pathophysiological mechanisms, or diagnostic developments. Case reports, editorials, commentaries, conference abstracts, non-English publications, and studies not relevant to the review objectives were excluded.

Titles, abstracts, and full texts were screened for relevance. Data regarding study design, population characteristics, investigated exposures, outcomes, and principal findings were extracted and synthesized narratively.

## 3. Global Epidemiology of Thyroid Cancer

PTC accounts for approximately 85–90% of all thyroid malignancies and is the histological subtype primarily responsible for the increasing incidence of TC observed worldwide [14,15].

### 3.1. Global Incidence Trends

Over the last thirty years, a gradual increase in TC incidence has been observed worldwide. In 2021, 249,538 new cases of this disease were reported, most of which occurred in women (167,237), while 82,301 cases were diagnosed in men. This confirms that TC is more common in the female population. During the analyzed period, the age-standardized incidence rate also increased from 2.062 per 100,000 people in 1990 to 2.914 in 2021. The average annual growth rate was 1.25, indicating a continuing increase in incidence [16].

Epidemiological changes are also visible at the level of individual countries. One example is China, where the number of new TC cases increased from 10,030 in 1990 to 39,080 in 2019, corresponding to an increase of nearly 290% [17]. Such a significant increase in incidence may indicate both the influence of environmental factors and greater availability of modern diagnostic methods. An increase in incidence is also observed in younger age groups. In the population of adolescents and young adults, 236,761 cases of TC were reported, including 63,509 in men and 173,252 in women [18]. As in the general population, a clear predominance of cases among women was also observed in this group. These data suggest that the increase in TC diagnoses does not only concern older individuals, but also includes increasingly broader age groups.

### 3.2. Geographic Variation

Depending on the region of the world and the socio-economic development level of a given country, differences in TC incidence can be observed. In countries with a higher level of social development, a greater number of malignant neoplasms, including TC, is more frequently observed. At the same time, countries with a lower social development index, despite a lower number of diagnoses, are characterized by higher disease-related mortality. In the analyzed data, TC ranked first in countries with a high social development index [18].

In both women and men, incidence rates were approximately five times higher in countries with high and very high levels of social development than in countries with low and medium levels of social development [19]. Additionally, in all regions, the highest incidence was observed among individuals over 70 years of age.

Similar findings were observed in studies concerning the level of socio-demographic development. In highly developed regions, the highest incidence of TC was reported, whereas the lowest incidence was observed in regions with a lower level of socio-demographic development in 2021.

In highly developed regions, increasing TC incidence rates were observed for many years, reaching their highest values around 2009. After this period, a gradual decline began to be noted. The greatest increase in the number of cases was observed in regions with a medium level of socio-demographic development [16]. Large differences in TC incidence are also visible between individual regions of the world. Incidence rates among women differed more than fifteenfold, and the highest values were recorded in Micronesia and French Polynesia, North America, and East Asia. A particularly high incidence of TC was observed in Republic of Korea, where the incidence rate among women was approximately 45 cases per 100,000 people [19].

Despite differences between world regions, an increase in the number of TC cases is observed in many countries, including the United States, China, and Canada. Saudi Arabia, the Republic of Korea, and Japan showed a higher average annual percentage increase in age-standardized incidence rates compared with countries with a similar level of socio-demographic development. In contrast, South Africa, Argentina, and Italy achieved lower values than the average [2].

Geographical differences are also visible within countries—for example, in the United States. Between 2001 and 2005, age-adjusted incidence rates ranged from 5.4 to 12.8 per 100,000 people depending on the state. The highest values were observed in areas including eastern Pennsylvania, New Jersey, and the southern part of New York State. Studies suggested that one of the possible factors influencing the increased incidence in this region may have been exposure to radioactive iodine emissions from nearby nuclear reactors [20].

Differences between ethnic groups were also observed in the United States. The highest incidence of TC was reported among non-Hispanic White individuals, followed by Asians and Pacific Islanders, Hispanics, and non-Hispanic Black individuals [14].

### 3.3. Age and Sex Differences

TC shows clear differences depending on sex and age. Global data indicate that the disease occurs significantly more often in women than in men. In 2021, 249,538 new cases were reported, of which 167,237 (67%) occurred in women and 82,301 (33%) in men [16]. Similar data have been observed in other analyses—in 2022, 236,761 cases were recorded among young adults, including 173,252 in women and 63,509 in men [18]. These differences are also confirmed by age-standardized incidence rates, which in 2020 were 10.1/100,000 for women and 3.1/100,000 for men [19].

Differences are also observed in the age at diagnosis. The highest number of new cases is observed in the middle-aged population. In 2021, the highest number of cases occurred in the 55–59 age group—11,441 cases in men and 19,601 in women. At the same time, the highest age-specific incidence rates were observed in women aged 70–74 years and in men aged 85–89 years [16], indicating that the risk increases with age.

Studies including longer observation periods show that the highest number of TC cases occurs among individuals aged 15–49 and 50–69 years, both in China and in G20 countries. In the 50–69 age group, an increase in the number of cases was observed, while at the same time the number of deaths decreased between 1990 and 2019. This may indicate better detection of the disease and more effective treatment. However, individuals over 70 years of age, despite a lower number of cases, had the highest mortality rate, which continued to increase during the analyzed period [17].

### 3.4. Histological Subtypes

TC includes several histological subtypes that differ in incidence, clinical course, and prognosis. The most common type is PTC, which accounts for nearly 90% of all TC cases. Although thyroid neoplasms occur rarely in children, PTC also remains the dominant histological type in this group and accounts for approximately 90% of malignant TC.

PTC is usually characterized by a good prognosis; however, several more aggressive variants of this tumor are also distinguished. These include the tall-cell, diffuse sclerosing, columnar, hobnail, and solid variants. These aggressive variants have been associated with increased recurrence rates, more invasive disease, and less favorable clinical outcomes despite the generally excellent prognosis associated with conventional PTC [15].

An additional factor that should be considered when interpreting recent trends in PTC incidence is the introduction of the non-invasive follicular thyroid neoplasm with papillary-like nuclear features (NIFTPs) category. This reclassification removed selected low-risk lesions from the category of papillary thyroid carcinoma and may therefore affect the interpretation of temporal trends in PTC incidence [21].

The second most common type of TC is follicular thyroid carcinoma (FTC), which originates from follicular cells producing thyroid hormones. It is estimated to account for approximately 10% of malignant thyroid tumors in areas with adequate iodine intake, whereas in iodine-deficient regions its proportion increases to 25–40%. Approximately 80% of FTC cases are characterized by a milder clinical course and good prognosis; however, the remaining cases show a more aggressive disease course. Three subtypes of FTC are distinguished: minimally invasive, encapsulated angioinvasive, and widely invasive. Compared with PTC, FTC more frequently gives distant metastases and is considered a malignancy with a more aggressive clinical course [15].

The 10-year overall survival rates for PTC and FTC are 93% and 85%, respectively, regardless of the stage of disease [22].

A less common type of thyroid malignancy is medullary thyroid carcinoma (MTC), which accounts for approximately 1–5% of all TC cases. It originates from parafollicular C cells responsible for calcitonin production. MTC may occur sporadically or as part of hereditary syndromes such as multiple endocrine neoplasia type 2A and 2B (MEN2A and MEN2B) and familial MTC. Most cases are sporadic, while approximately 25% are associated with MEN2 syndrome. This type of cancer has a less favorable prognosis than PTC and FTC because of its greater tendency to form metastases and later diagnosis of the disease.

The rarest—and at the same time, the most aggressive—type of TC is anaplastic thyroid carcinoma (ATC). It accounts for approximately 2% of all thyroid neoplasms, but is responsible for about half of the deaths related to malignant tumors of this organ. ATC is an undifferentiated malignancy that microscopically shows complete loss of follicular differentiation and lack of diversity in cellular structure. Due to its highly aggressive course and rapid metastasis formation, the prognosis for this type of cancer remains very poor [15].

In a study conducted in France, including data from six cancer registries from 1983 to 2000, 3851 TC cases were analyzed. Most diagnoses concerned women—3020 cases were recorded, representing 78.4% of all cases. It was demonstrated that the most common histological subtype was PTC, which accounted for 2769 cases, representing 71.9% of all diagnosed TC. The authors noted that the increase in TC incidence observed in Western countries mainly concerned PTC, while the incidence of other histological types remained relatively stable or showed a decreasing tendency. In each analyzed region, the highest average annual rates of change were observed for the smallest tumors, suggesting that the increase in the number of diagnoses was particularly associated with the detection of small papillary lesions [23].

### 3.5. Tumor Characteristics at Diagnosis

Studies on TC have shown that tumors smaller than 10 mm in diameter were the most frequently diagnosed and accounted for 45.9% of all cases. Lesions measuring 10–20 mm accounted for 24.1% of diagnoses, whereas tumors with a diameter of 20–40 mm constituted 19.5% of cases. Neoplasms larger than 40 mm occurred less frequently and accounted for 5.4% of cases. In some analyses (6.9%), tumor size was not specified. These results show that diagnoses of small thyroid lesions have predominated in recent years [23].

Tumor size is also associated with patient prognosis. In PTC, better treatment outcomes were observed in patients with tumors smaller than 1.5 cm. Similar findings were also observed in FTC, where smaller lesions (<1.5 cm) were associated with a more favorable disease course [15].

At the same time, it has been suggested that the increase in incidence does not concern only small and localized tumors. The greatest increase in the number of diagnoses was indeed observed for tumors ≤1 cm; however, an increased incidence was also reported for more advanced PTC and tumors larger than 4 cm [14].

These findings suggest that better detection alone may not be enough to fully explain current epidemiologic trends. The concurrent rise in the number of larger and more advanced tumors indicates that the change in environmental, metabolic or lifestyle-related risk factors may also contribute to the increase in incidence.

### 3.6. Incidence–Mortality Paradox

In the epidemiology of TC, attention is drawn to the phenomenon in which the number of new cases continues to increase, while mortality rates remain relatively low and stable. This means that despite the growing number of diagnosed TC cases, the number of deaths related to this disease does not increase to the same extent [14]. This relationship is referred to as the “incidence–mortality paradox” and has been an important topic of epidemiological analyses for many years. Although this divergence is commonly presented as evidence for overdiagnosis, other explanations should also be considered. These include lead-time bias, length bias, improvements in treatment outcomes, demographic aging, changes in disease classification, and differences in completeness of cancer registry between countries and regions.

Epidemiological data indicate that mortality caused by TC remains low. Age-standardized mortality rates were approximately 0.5 per 100,000 women and 0.3 per 100,000 men. In most analyzed countries, the number of deaths related to TC did not exceed one case per 100,000 individuals, regardless of sex. At the same time, some countries showed a very large difference between the number of new cases and the number of deaths. The highest incidence-to-mortality ratio was observed in Republic of Korea, followed by Cyprus and Canada [19].

Similar relationships were also described in the population of young adults. In 2022, 236,761 TC cases were recorded worldwide in this age group, whereas the number of deaths was only 2107 [18]. These data indicate that despite the increasing number of diagnoses, TC-related mortality remains relatively low.

Since the 1970s, an increase in incidence accompanied by stable mortality has been observed in most regions of the world, including the United States, Canada, Europe, Australia, Asia, and some countries in South America. For example, in Republic of Korea, TC screening—which rapidly became widespread—led to a sudden increase in the number of diagnosed cases. Between 1993 and 2011, TC incidence increased approximately fifteenfold, mainly due to the increased detection of PTC, while mortality rates remained relatively stable [14].

The increasing incidence and relatively stable mortality provide evidence consistent with the notion that overdiagnosis plays an important role in contemporary TC epidemiology. The simultaneous rise in larger and more advanced tumors suggests that overdiagnosis alone is unlikely to explain all of the observed trends. The relative contribution of diagnostic practices and real changes in disease occurrence remains an area of ongoing investigation.

## 4. Reassessing Overdiagnosis

### 4.1. Definition and General Mechanisms of Overdiagnosis

In recent years, increasing attention has been drawn to the problem of TC overdiagnosis. More advanced diagnostic methods and the more frequent use of imaging examinations have resulted in the detection of a greater number of small and asymptomatic thyroid lesions that previously might not have been diagnosed. Overdiagnosis refers to the diagnosis of a disease or abnormality that would not cause symptoms or negatively affect a patient’s health during their lifetime if it had remained undetected. Initially, this phenomenon was mainly associated with cancer screening programs; however, it is now emphasized that it may also result from diagnostic examinations performed in individuals without clinical symptoms [24].

This phenomenon has mainly been observed in regions with high medical resources. It is estimated that almost half of TC cases in men and more than 80% of cases in women in these regions may represent overdiagnosis, corresponding to more than 500,000 cases. Many factors contribute to the development of this problem, including increased use of advanced diagnostic technologies, financial incentives, medical culture promoting more frequent examinations and treatment, limitations in scientific evidence, ineffective screening programs, and the expansion of disease definitions [24].

TC overdiagnosis mainly results from the increasing availability of diagnostics and the more frequent detection of lesions that might never have had clinical significance. This phenomenon shows that an increase in the number of diagnoses does not always indicate a true increase in the number of clinically significant cancers.

### 4.2. Contribution of Diagnostic Imaging

The development of imaging methods has had a significant impact on the increase in TC diagnoses in recent years. Thyroid ultrasonography is the primary examination used in the diagnosis of this organ [25].

The increasingly frequent use of ultrasonographic examinations has led to more frequent detection of small and asymptomatic thyroid lesions, including small cancers incidentally diagnosed in patients without clinical symptoms. It has also been shown that residents of regions where thyroid ultrasonography is performed more frequently are diagnosed with TC more often than individuals living in regions with lower use of this examination. Despite data indicating a relationship between ultrasonography and the detection of low-risk cancers, the number of performed examinations continues to increase gradually, and the growth rate is estimated at approximately 20% annually [26].

Other imaging methods, such as CT, MRI, and PET, are also becoming increasingly important in diagnostics. Mortality rates remain unchanged; however, the more frequent use of these examinations increases the number of incidentally detected thyroid lesions and TC diagnoses [24]. Nevertheless, the results of some studies suggest that the number of incidentally detected nodules may be lower than previously thought. In a retrospective analysis conducted at a center in Chicago, including approximately 98,000 head and neck imaging examinations, incidentally detected thyroid nodules accounted for only 0.4% of all reported lesions [14].

### 4.3. Incidentally Detected Thyroid Nodules

Incidentally detected thyroid nodules, referred to as incidental thyroid nodules, are defined as clinically non-palpable lesions that differ radiologically from the surrounding thyroid parenchyma and are detected during imaging examinations performed for reasons unrelated to thyroid disease diagnostics. The occurrence of these lesions is very common. Autopsy studies indicate that thyroid nodules may be present in up to 50–60% of adults. Incidental thyroid nodules are detected in many imaging examinations. It is estimated that they are present in 20–67% of ultrasonographic examinations, up to 25% of contrast-enhanced chest CT scans, 16–18% of MRI examinations, and 1–2.3% of PET examinations. The main aim of further diagnostics is to distinguish benign lesions from malignant neoplasms [27].

In individuals without a history of external beam radiation exposure and without a family history of MTC, the risk of malignancy of a nodule detected during imaging examinations such as ultrasonography, CT, or MRI is estimated at 5–13% [28].

Studies indicate that approximately 7% of incidentally detected thyroid nodules are ultimately diagnosed as TC based on FNAB. This value is similar to the malignancy rate observed in nodules detected during standard ultrasonographic examinations. At the same time, results from studies conducted in Chicago suggest that incidentally detected thyroid nodules in routine clinical practice may occur less frequently than previously indicated by analyses based on detailed radiological assessment.

Analysis of incidentally detected lesions according to tumor size showed that tumors with a diameter of ≤10 mm were detected incidentally in approximately 50% of cases, whereas tumors larger than 10 mm were detected incidentally in only 29% of cases. The frequency of incidental detection was slightly higher in men than in women. In addition, histological analysis demonstrated that incidentally detected neoplasms were most commonly small PTC.

The study also found that approximately 49% of TC cases were detected incidentally in asymptomatic individuals during histological or imaging examinations, particularly during neck ultrasonography, which accounted for 27% of such diagnoses. Most of these lesions were PTC, accounting for approximately 76% of cases [29].

### 4.4. Screening and the South Korean Experience

TC screening may enable earlier detection of malignant lesions. This may improve treatment effectiveness and reduce the risk of later complications. Screening diagnostics are mainly based on neck palpation, ultrasonography, or a combination of both methods. However, it is emphasized that screening may lead to overdiagnosis through the detection of very small or slow-growing tumors. Such tumors would probably not cause clinical symptoms or affect the patient’s life expectancy [22].

So far, randomized controlled trials have not confirmed the effectiveness of TC screening. Moreover, studies evaluating intermediate endpoints, such as changes in tumor stage at the time of diagnosis, have not been conducted. Contrary to common belief, population-based TC screening programs do not operate in the United States, the United Kingdom, or most European countries, despite the incidental detection of neoplastic lesions during routine neck palpation examinations. In these countries, an increase in TC incidence has been observed while mortality rates remained stable or slightly decreased [30].

Preventive healthcare covers a significant part of the population and may have serious health and social consequences, which require investigation of the problem of overdiagnosis. Excessive use of screening may lead to unnecessary diagnoses and treatment in patients who would probably never experience the effects of the disease [31].

The situation in Republic of Korea is one of the most frequently described examples of the impact of screening on the increase in TC diagnoses. Between 1993 and 2011, the number of TC diagnoses increased approximately fifteenfold after the introduction of a free national screening program in 1999. However, there were no significant changes in disease-related mortality. Due to growing concerns regarding excessive diagnosis and treatment, screening practices began to be limited in subsequent years. In 2015, the Korean Committee for National Cancer Screening Guidelines stated that ultrasonographic TC screening should not be performed in healthy individuals without clinical symptoms [30]. However, the Korean experience is representative of a unique healthcare environment with widespread and opportunistic screening and an intensive use of ultrasonography. Therefore, caution is warranted when generalizing these results to other high-income countries with different healthcare systems and diagnostic practices.

The literature also emphasizes the role of diagnostic thresholds in screening. A low diagnostic threshold leads to the diagnosis of a greater number of patients, some of whom may be overdiagnosed. On the other hand, a higher diagnostic threshold is associated with fewer cases of overdiagnosis and fewer harms related to treatment. In such situations, the benefits of disease detection may be limited, while the risk of unnecessary treatment remains important [24].

TC screening may increase the detection of early-stage neoplasms, but it is also associated with the risk of overdiagnosis and overtreatment. Available data suggest that the widespread use of screening, especially ultrasonography in asymptomatic individuals, may increase the number of diagnoses without a significant impact on disease-related mortality. Currently, however, there is no clear evidence confirming the benefits of population-based screening for TC detection.

### 4.5. Evidence Supporting and Challenging the Overdiagnosis Hypothesis

Epidemiological evidence supports a substantial contribution of overdiagnosis to the increasing incidence of TC. Increased use of ultrasonography and other imaging modalities has resulted in the more frequent detection of small, asymptomatic thyroid lesions, many of which are diagnosed incidentally [14,24,26,27,29].

The continued presence of relatively stable mortality rates despite rising incidence further supports the overdiagnosis hypothesis. This trend was particularly evident in Republic of Korea after widespread use of ultrasonography-based screening [14,19,30].

However, the research that is now available indicates that overdiagnosis may not be the only explanation for the rising prevalence of TC. Higher incidence of bigger tumors and more advanced disease stages have been reported in a number of studies; these findings are hard to explain by the better detection of small indolent lesions.

According to epidemiological studies, the incidence of PTC larger than 4 cm, as well as tumors at more advanced stages of the disease, has also increased. If the increase in incidence resulted solely from overdiagnosis, a rise mainly in the number of small, incidentally identified tumors would be expected. However, the observed increase in larger and more aggressive neoplasms suggests that there may also be a true increase in disease occurrence. Nevertheless, overdiagnosis may account for approximately 50% of the increase in PTC cases [14].

The rise in TC incidence in different age groups and geographical areas, and the increasing prevalence of obesity, environmental exposures and other potential risk factors, suggest that other causes other than overdiagnosis may also play a part in current epidemiological trends [14,17,18,20].

Overdiagnosis is widely acknowledged as a significant factor in the rising incidence of TC, but its relative impact is likely to differ by populations and healthcare systems. In highly screened populations such as Republic of Korea, there is evidence to suggest that overdiagnosis may be responsible for a significant proportion of newly diagnosed cases. However, the increase in the incidence of larger and more advanced tumors suggests that the changes in the underlying risk factors may also be contributing to the current epidemiological trends. Thus, contemporary patterns of TC incidence are likely to be formed both by overdiagnosis and by a real increase in disease occurrence.

## 5. Advances in Diagnostic Technologies

In the past, thyroid nodules were detected incidentally through physical examination and palpation, whereas in contemporary clinical practice the increased use of routine diagnostic tests, including ultrasound (US), has led to a notable rise in the detection rate of TC. Furthermore, changes in guidelines and clinical practice are leading to an increase in the number of diagnoses, including in cases of asymptomatic tumors [28,32,33,34].

High-frequency ultrasound is a widely used diagnostic tool for TC due to its non-invasive nature, ease of use, low cost and reproducibility. Technological advances have improved the diagnostic performance of ultrasound and significantly expanded the capabilities of ultrasound through new techniques such as contrast-enhanced ultrasound (CEUS), which allows for the assessment of microcirculation in nodules, and elastography, which enables the assessment of tissue stiffness. Ultrasound enables the detection and characterization of thyroid nodules and allows for the detection of small and subtle changes and the assessment of their size, shape, margins, echogenicity, calcifications and vascularization. However, its diagnostic accuracy remains operator-dependent. The accuracy of this examination depends on the size of the nodule, its location, histological type and the operator’s experience, and limitations in resolution may lead to diagnostic errors. The morphological similarity between benign and malignant lesions complicates diagnosis due to the lack of clear standards for interpretation [34].

Fine-needle aspiration biopsy (FNAB) enables minimally invasive, rapid and relatively inexpensive diagnosis of thyroid nodules, which makes it widely used in clinical practice. Ultrasound features suggestive of malignancy are used to determine eligibility for FNAB. The implementation of malignancy is used to determine eligibility for FNAB. The implementation of ultrasound-based risk stratification systems has improved the selection of nodules for biopsy and reduced unnecessary invasive procedures. This technique enables the early detection of TC amongst numerous benign nodules, which also leads to a more precise selection of patients for treatment. Due to its ease of access and simplicity, FNAB contributes to an increase in the number of diagnosed cases of TC, which is significant in the context of analyzing incidence trends [35]. Although FNAB remains a widely used and clinically valuable diagnostic tool for the evaluation of thyroid nodules, false-negative, false-positive and indeterminate results may occur. Cytological uncertainty remains an important diagnostic challenge and has stimulated the development of complementary molecular testing strategies [36,37]. The increase in the number of ultrasound scans correlates with a rise in the number of TC diagnoses due to the greater sensitivity of ultrasound compared with a physical examination, which has a low detection rate for nodules; for nodules larger than 2 cm, the detection rate on palpation was only 48%. Furthermore, the introduction of ultrasound criteria for FNAB eligibility has led to an increase in the number of biopsies, which has contributed to an increase in the diagnosis of asymptomatic tumors [32].

In cases of indeterminate cytology, molecular diagnostics has emerged as an important adjunct to FNAB and may help refine malignancy risk assessment and clinical decision-making. Advances in our understanding of the molecular mechanisms underlying TC have enabled the identification of genetic markers that aid diagnosis. Molecular testing of FNAB samples can improve the differentiation between benign and malignant lesions. In FNAB diagnostics, analysis of somatic mutations such as *BRAF*, *RAS*, and *RET/PTC* and *PAX8*/Peroxisome Proliferator-Activated Receptor Gamma (*PPARγ*) rearrangements, as well as gene expression and microRNA classifiers, is employed. Material obtained during FNAB can be stored and later used for molecular testing. Immunocytochemical markers have not achieved sufficient diagnostic accuracy, and molecular diagnostics aims to increase the sensitivity and specificity of distinguishing between benign and malignant lesions [38].

The introduction of ultrasound risk stratification systems, such as the American Thyroid Association (ATA) classification and Thyroid Imaging Reporting and Data System (TI-RADS), has improved the standardization of nodule assessment and FNAB selection. Similarly, molecular classifiers such as Afirma (Veracyte, Inc., South San Francisco, CA, USA) and ThyroSeq (CBLPath, Inc., Rye Brook, NY, USA) have enhanced the evaluation of indeterminate FNAB results, reducing diagnostic uncertainty and improving risk stratification [39].

Modern diagnostic tools have improved the ability to identify subclinical forms of cancer, including papillary microcarcinomas detected incidentally. Much of the increase in thyroid cancer detection has been attributed to opportunistic ultrasound examinations and incidental findings during imaging performed for unrelated clinical indications. These technologies enable earlier detection of the disease at the pre-symptomatic stage, altering the profile of diagnosed cases in the population. At the same time, increased diagnostic sensitivity translates into the detection of lesions that would not present clinical symptoms without the use of advanced imaging methods, which is of significant importance in the analysis of epidemiological trends [34].

The evolution of TC staging systems has resulted in the reclassification of patients between stages based on tumor and lymph node characteristics, contributing to stage migration. In parallel, there has been a shift in clinical interpretation toward recognizing low-volume nodal disease and adopting less aggressive management strategies, reflecting diagnostic drift in the assessment of TC [40].

In summary, the evolution of diagnostic technologies, particularly high-resolution ultrasound, have substantially increased the detection of clinically silent thyroid nodules and early-stage thyroid cancers. The integration of ultrasound-based risk stratification, FNAB, and molecular testing has improved diagnostic accuracy and patient selection while also contributing to the increased detection of subclinical disease.

## 6. Obesity and Metabolic Factors

Obesity is one of the most important modifiable risk factors for many cancers, including TC. The increasing prevalence of obesity and the rising incidence of thyroid cancer suggest a potential association between these phenomena [12,41,42,43]. Studies including populations from the USA, Japan, Canada, and many other countries suggest a positive association between higher body mass index (BMI) and the risk of thyroid cancer [12,42]. Analyses of subgroups including age, obesity indicators, and geographical region, mainly in Europe and Asia, also showed that the association between obesity and TC development was present in all analyzed groups, which suggests that the observed association is relatively consistent across the studied populations [12,43]. Different classes of metabolic obesity are distinguished, including individuals with normal body weight without metabolic disorders, individuals with normal body weight but with metabolic disorders, individuals with obesity but without features of metabolic disorders, and individuals with obesity and coexisting metabolic disorders [41,42,43]. In a cohort study including 255,051 adults, it was shown that the risk of TC was 1.47 (95% CI: 1.12–1.93) in metabolically healthy individuals with obesity and 1.26 (95% CI: 1.03–1.53) in metabolically unhealthy individuals with obesity compared with individuals with normal body mass index [41]. Another study including different obesity phenotypes in relation to the risk of breast, colorectal, endometrial, ovarian, pancreatic, liver, gallbladder, kidney, and TC suggest that metabolic disorders may be equally important or even more important than body weight itself [41,42,43]. An unfavorable class of metabolic obesity may increase the risk of cancers even in individuals who are not overweight. However, in the case of obesity classes without metabolic disorders and with coexisting metabolic disorders, the observed increase in risk did not always reach statistical significance, which indicates the complex nature of the relationship between obesity, metabolic disorders, and carcinogenesis [41]. One should also consider the possibility of detection bias in the interpretation of the association between obesity and thyroid cancer—associated with metabolic diseases and other obesity-related disorders—which are associated with a more frequent use of healthcare services and diagnostic examinations. lead to more incidental detection of thyroid nodules and thyroid cancer. Thus, some of the obesity–thyroid cancer association we observed may be due to greater detectability of neoplastic lesions in people subject to more frequent diagnostic evaluation [44].

Obesity is associated with a number of metabolic and hormonal disorders that may mediate the increased risk of cancers, including TC. Among the most important mechanisms are insulin resistance, hyperinsulinemia, chronic inflammation, and disorders of adipokine secretion [45,46,47,48,49]. Adipose tissue as a whole performs not only the function of an organ storing fatty acids, but also the function of an endocrine tissue [45,50].

Adipokines, which are signaling proteins, influence various biological processes. They regulate metabolism, insulin sensitivity, and the body’s inflammatory response [45,50]. In obese individuals, as a result of tissue hypoxia, adipocyte secretion changes toward a proinflammatory profile, which promotes the persistence of a chronic systemic inflammatory state [45,46,47,48,49]. This leads to the release of proinflammatory and prooncogenic mediators from adipose tissue, which may promote the formation and development of cancers in various organs, including the thyroid gland [45,46,47,48,49]. Adipokines include—among others—leptin, adiponectin, chemerin, resistin, omentin-1, nesfatin, visfatin, and dipeptidyl peptidase 4 (DPP-4) [45,50].

**Molecular pathways and potential impact of major adipokines on TC in the context of obesity.**

**A**
**dipokine**

**Main Receptor**

**Main Signaling Pathways**

**Concentration in Obesity**

**Potential Impact on TC**

**References**
LeptinOb-RActivation of JAK2/STAT3, PI3K/AKT and ERKIncreasedProliferation epithelial–mesenchymal transition, tumor size, lymph node metastasis[12,13,16,17]AdiponectinAdipoR1 and AdipoR2Activation of AMPK and inhibition of mTORDecreasedAntiproliferative and proapoptotic effects[12,13,16,17]ChemerinCMKLR1, GPR1, CCRL2Activation of PI3K/Akt and MAPKIncreasedPromotion of inflammation, insulin resistance[12,13]ResistinRETNActivation of JNK and p38 MAPKIncreasedInsulin resistance, chronic inflammation[12,13]Omentin-1Receptor has not yet been clearly identifiedActivation of Akt and AMPKDecreasedAnti-inflammatory effects[12,13]Nesfatin-1Has not been clearly identifiedInhibits MAPK, including p38 MAPK/c-Jun and IKK/NF-κBDecreasedAnti-inflammatory and antioxidant effects[12,13]VisfatinNo specific receptorIncreased hTERT expressionIncreasedMay contribute to a more invasive tumor phenotype, proliferation of cancer cells[12,13]Dipeptidyl Peptidase-4 (DPP-4)Not clearly establishedDecreased Akt phosphorylationIncreasedInsulin resistance, chronic inflammatio[12,13]


The relationship between obesity and the increased risk of TC may be mediated by metabolic disorders, among which insulin resistance plays a key role [51,52]. Insulin resistance develops as a result of the interaction between genetic and environmental factors [51,53]. Inherited forms of insulin resistance and diabetes are associated with mutations affecting the insulin receptor (INSR) or other elements of the insulin signaling pathway [53]. However, the main factor in the development of insulin resistance is the excessive accumulation of visceral fat resulting from a sedentary lifestyle and excess calorie intake [51,54]. Most obese individuals develop impaired insulin action, although peripheral insulin sensitivity often improves after weight reduction [51]. The insulin receptor additionally shapes signaling effects. INSR-B, as a metabolic receptor, mediates insulin-dependent glucose uptake in target tissues. INSR-A has a greater affinity for insulin, therefore it undergoes processing more rapidly, and its role is also to bind proinsulin and insulin-like growth factors (IGFs) [52,53]. INSR-A is produced in increased amounts in malignant tumors due to its role in mitogenic signaling [52,53]. In obesity, insulin resistance is initially compensated by hyperinsulinemia [53]. Chronic excess insulin continuously stimulates the insulin/IGF axis, particularly through INSR-A, which is highly produced in several malignant tumors, including PTC [52,53,55]. Activation of the IGF-1R receptor may enhance the growth and proliferation of cancer cells [52,53]. The PI3K/AKT/mTOR and MAPK/ERK pathways, through cell cycle progression, resistance to apoptosis, and angiogenesis, play an important role in oncogenic activity [52,53,55]. TC with an autocrine IGF2/INSR-A loop may support the proliferation of thyroid cancer cells independently of TSH [53,55]. Hyperinsulinemia also disrupts the cell cycle through induction of cyclin D1 and inhibition of tumor suppressor pathways [53]. This potentially suggests that hyperinsulinemia is a factor intensifying thyrocyte proliferation and nodule formation. Therefore, hyperinsulinemia may represent one of the potential mechanisms linking obesity to the development of thyroid cancer [52,53,56,57].

## 7. Environmental Exposures

Environmental factors have been proposed as contributors to thyroid carcinogenesis, although the strength of evidence varies considerably between exposures, with ionizing radiation representing the most firmly established risk factor. Increasing attention has also been directed toward air pollution, heavy metals, and endocrine-disrupting chemicals as potential contributors to thyroid cancer [58,59].

### 7.1. Air Pollution

Air pollution is considered a worldwide recognized cancer risk factor, which increases the production of reactive oxygen species (ROS), leading to oxidative DNA damage, lipid peroxidation, mitochondrial dysfunction and genomic instability. Air pollutants induce chronic low-grade inflammation, activate inflammatory cytokines and create a pro-tumorigenic microenvironment [60]. Particulate Matter (PM) is a complex mixture of airborne particles, with PM2.5 capable of entering systemic circulation and exerting systemic biological effects [58,59]. From a mechanistic perspective, the thyroid may be especially vulnerable to PM2.5 exposure because thyroid hormone production relies on tightly regulated redox reactions and endocrine feedback mechanisms. PM2.5 can carry toxic compounds such as metals and EDCs, which may impair thyroid function by increasing oxidative stress, promoting inflammation, and interfering with endocrine signaling pathways [61]. Among the examined air pollutants, PM2.5 has shown the most consistent association with TC risk, with epidemiological studies generally reporting modest increases in risk. A large population-based study demonstrated a positive association between long-term PM2.5 exposure and thyroid cancer incidence, with evidence of a dose–response relationship, supporting the hypothesis that chronic exposure to ambient air pollution may contribute to thyroid carcinogenesis [9]. However, the strength of the association varies across studies, likely reflecting differences in exposure assessment, study populations and environmental conditions, highlighting the need for further prospective investigations [10]. Some studies suggest that higher PM2.5 concentrations and longer exposure durations may be associated with increased TC risk. PM2.5 particles penetrate deep into the lungs, can enter the bloodstream, cause systemic inflammation and exacerbate oxidative stress [60]. Recent evidence from systematic analyses further supports an association between exposure to particulate air pollution—particularly PM2.5—and increased thyroid cancer risk, although heterogeneity between studies remains substantial [62]. Observational studies reported an increased incidence of TC among workers involved in the World Trade Center rescue and recovery operations after the September 11 attacks. However, interpretation of these findings is complicated by intensive medical surveillance of this population and potential exposure to multiple carcinogenic agents beyond PM alone [63]. Despite growing evidence supporting a link between PM2.5 exposure and thyroid cancer risk, most available data originate from observational studies and should be interpreted cautiously. Residual confounding, differences in healthcare utilization, diagnostic intensity, socioeconomic factors and exposure assessment methods may influence reported associations. Therefore, further prospective studies with improved exposure characterization and control of potential confounders are required to clarify the causal role of air pollution in thyroid carcinogenesis [9,62].

### 7.2. Ionizing Radiation

Ionizing radiation is the best-documented environmental risk factor for TC, particularly following exposure during childhood and adolescence. The thyroid gland is exceptionally sensitive to ionizing radiation, especially in children, due to the rapid proliferation of thyrocytes in early childhood and higher mitotic activity before the age of five. Radiation causes single- and double-strand breaks in DNA, induces chromosomal rearrangements and generates ROS that damage genetic material. The highest risk applies to infants and young children, whilst the radiosensitivity of the thyroid gland decreases significantly after the age of 15–20. The risk of TC increases linearly with increasing radiation dose from 0.05–0.1 Gy to approximately 20–29 Gy. At doses above 30 Gy, a partial decrease in risk is observed, likely due to the radiation-induced cell-killing effect [64,65]. One of the most important epidemiological sources of evidence is the Life Span Study of atomic bomb survivors from Hiroshima and Nagasaki, which demonstrated a clear dose-dependent increase in thyroid cancer incidence, particularly among individuals exposed at a young age [66]. Evidence linking ionizing radiation and thyroid cancer comes from both accidental environmental exposures and medical or occupational sources. Environmental sources of ionizing radiation include the Chernobyl disaster, where huge quantities of radioactive iodine were released, and factors exacerbating exposure included delayed evacuation, iodine deficiency, the lack of iodine prophylaxis and the absence of dietary restrictions, which resulted in the first cases of TC as early as four years after the disaster. The Fukushima accident, in contrast, occurred under conditions of rapid evacuation, dietary restrictions and higher dietary iodine intake, which resulted in significantly lower thyroid doses compared to Chernobyl and no clinically detectable incidence of TC within the first decade of follow-up [64]. Large case–control and cohort studies conducted in Belarus, Ukraine and Russia following the Chernobyl accident confirmed a marked increase in thyroid cancer incidence among individuals exposed in childhood, with a latency period of approximately 4–5 years and the highest risk observed in the youngest age groups at exposure [67]. Follow-up studies of the Fukushima Daiichi nuclear accident population, including analyses from the Fukushima Health Management Survey, have not demonstrated a clear increase in thyroid cancer incidence attributable to radiation exposure during the first decade after the accident, although ongoing surveillance continues [68]. Medical radiation is an increasingly important source of exposure, particularly due to the development of CT imaging, fluoroscopy, nuclear medicine and radiotherapy. The growing use of imaging techniques is increasing the cumulative radiation dose, particularly in children and young adults. CT scans are among the main contributors to medical radiation exposure, particularly in pediatric populations. This association has been investigated in several large epidemiological cohort studies based on national health databases, which aimed to quantify cancer risk associated with cumulative diagnostic radiation exposure in childhood. Evidence from large population-based cohort studies using national health databases in the United Kingdom, the United States and Australia has shown an association between cumulative diagnostic radiation exposure in childhood and an increased risk of cancer, including thyroid malignancies, with computed tomography identified as a major contributor to medical radiation burden in pediatric populations [69]. However, it should be emphasized that diagnostic doses are significantly lower than those following nuclear accidents. Studies on diagnostic radiation suggest a small to moderate increase in thyroid cancer risk, with variability across exposure types and study populations [64]. While ionizing radiations are firmly known as carcinogens, the non-ionizing radiation (NIR), including those produced by mobile phones, are not yet fully understood. Evidence linking non-ionizing radiation from mobile phones to thyroid cancer remains inconsistent and inconclusive [59].

### 7.3. Heavy Metals

Heavy metals have emerged as a potential environmental risk factor in thyroid carcinogenesis, supported by growing epidemiological and experimental evidence [8]. Epidemiological studies suggest associations between exposure to heavy metals and altered thyroid function, as well as increased risk of thyroid malignancies, although causality has not been definitively established [70]. Metals such as cadmium, arsenic, lead, mercury, and nickel are most frequently investigated in the context of thyroid cancer due to their endocrine-disrupting properties [71]. Volcanic and geothermal areas represent specific environmental settings where increased exposure to arsenic, mercury, and other trace metals may occur through contamination of soil, water, and the food chain [8,72]. Experimental evidence indicates that heavy metals may promote thyroid carcinogenesis through the induction of oxidative stress and ROS, leading to DNA damage and genomic instability [70,73]. In addition, heavy metals may interfere with thyroid hormone synthesis and iodine uptake, contributing to dysregulation of the hypothalamic–pituitary–thyroid axis [71]. Some metals have also been shown to activate oncogenic signaling pathways involved in cell proliferation and apoptosis resistance in thyroid follicular cells [74]. Emerging evidence suggests that heavy metal exposure may also induce epigenetic alterations, including changes in DNA methylation and microRNA expression, which are increasingly recognized in thyroid tumorigenesis [8]. Although overdiagnosis contributes significantly to the observed increase in thyroid cancer incidence, studies from high-exposure environmental regions suggest that this phenomenon cannot fully explain observed geographic and temporal patterns [72]. Recent reviews indicate that evidence for individual heavy metals remains heterogeneous, and that combined or chronic low-dose exposure may be more relevant than single-metal effects in thyroid carcinogenesis [73].

### 7.4. Endocrine-Disrupting Chemicals

EDCs are environmental contaminants capable of interfering with thyroid function and hormone regulation [58,75]. Multiple studies have indicated that exposure to EDCs may interfere with normal thyroid function and contribute to a higher risk of adverse health effects, including developmental disturbances, thyroid diseases, and different forms of cancer [59,75]. However, long-term studies about the thyroid-related outcomes of ECDs are still lacking [58]. The action of these molecules is not linked to a reduction in thyroid hormone levels, but rather to the disruption of the biosynthesis and transport mechanisms of these hormones [59]. Phthalates are widely used plasticizers that may interfere with endocrine signaling pathways and thyroid hormone regulation. Phthalates may interfere with endocrine signaling pathways, potentially affecting thyroid hormone regulation and cellular proliferation. Phthalates may interfere with thyroid hormone signaling pathways, although their role in thyroid carcinogenesis has not been clearly established. Bisphenol A (BPA) may disrupt thyroid hormone synthesis, secretion and receptor-mediated signaling. Although experimental studies have identified several mechanisms through which BPA may interfere with thyroid function, evidence from human studies remains limited and inconclusive [58,59,75]. Polychlorinated biphenyls (PCBs) are persistent environmental pollutants that may interfere with thyroid hormone metabolism and receptor-mediated signaling. Experimental studies suggest that PCBs may influence pathways involved in thyroid carcinogenesis, while occupational studies have reported a possible association between PCB exposure and thyroid cancer. However, the available evidence remains insufficient to establish causality [59]. Overall, epidemiological evidence suggests a potential association between exposure to selected endocrine-disrupting chemicals and thyroid dysfunction or thyroid cancer; however, results remain inconsistent and heterogeneous across studies, and causality has not been established. The interpretation of available evidence is limited by heterogeneity in study design, variability in exposure assessment, and the predominance of observational studies. A summary of the main classes of endocrine-disrupting chemicals, their principal sources of exposure, and their proposed thyroid-related effects is presented in Table 1.

## 8. Molecular and Genetic Drivers of Thyroid Cancer

TC formation is a result of a combination of genetic alterations, epigenetic changes, and molecular pathways influenced by environmental factors. Together they dictate tumor initiation, progression, and clinical behavior.

### 8.1. Crucial Oncogenic Drivers

The molecular hallmarks of TC are alterations involving the MAPK and PI3K/AKT/mTOR signaling pathways.

#### 8.1.1. *BRAF* V600E Mutation

The *BRAF* V600E gene mutation is the most frequent genetic alteration in patients with PTC. It is present in approximately 45% of cases [76,77,78]. This mutation causes a constitutive activation of the MAPK signaling cascade, leading to a constant activation of mitogen-activated protein kinase kinase (MEK) and ERK proteins, enhancing uncontrolled cellular proliferation, migration, and invasion [76,78,79]. A hallmark of *BRAF* V600E-driven tumors is their ability to activate the MAPK pathway via disruption of the negative feedback regulation from ERK to RAF, which magnifies oncogenic signaling and transcriptional changes [76]. Molecular alterations also involve the downregulation of genes involved in iodine uptake and thyroid hormone synthesis, such as sodium/iodide symporter (*NIS*) gene, resulting in reduced thyroid differentiation and decreased sensitivity to radioactive iodine (RAI) therapy [76,79]. *BRAF* V600E mutation is clinically characterized by more aggressive tumor behavior, extrathyroid extension, lymph node metastases, and recurrence [76,77,78]. Moreover, it was found to be associated with poor clinical prognosis even in small PTC (≤1.5 cm) and papillary thyroid microcarcinoma (PTMC) (≤1 cm) [80].

#### 8.1.2. *RAS* Gene Family

Mutations of Neuroblastoma RAS Viral Oncogene Homolog (*NRAS*), Harvey Rat Sarcoma Viral Oncogene Homolog (*HRAS*), and Kirsten Rat Sarcoma Viral Oncogene Homolog (*KRAS*) genes are among the most frequent genetic alterations in TC and are especially enriched in follicular patterned thyroid tumors [81,82]. Their frequencies vary across different types of TC, being relatively high in FTC (41%), follicular variant papillary thyroid carcinoma (FVPTC) (55%), and encapsulated FVPTC (48%), but lower in classical PTC (24%) and medullary thyroid carcinoma (MTC) (15%) [81]. In the *RAS* family, the most affected gene is *NRAS* (69%), followed by *HRAS* (26%) and *KRAS* (7%). The most common genetic alteration associated with these mutations is a substitution of codon 61 in *NRAS*, leading to an amino acid change from glutamine to arginine (Q61R) [81,82]. *RAS* mutations are functionally involved in oncogenicity via activation of MAPK and PI3K/AKT signaling pathways, which are important for thyroid tumor development and progression [81]. *RAS* mutations have been identified in benign thyroid nodules like follicular adenoma as well as in well-differentiated carcinomas [81,82,83]. Regardless of their carcinogenic potential, the presence of a *RAS* mutation cannot be considered as a definitive sign of cancer, and consequently they are usually classified as indeterminate in cytological tests. The malignancy rate of *RAS*-mutant thyroid nodules is believed to be between medium and high, with the estimates between 29% and even 80–90% [82,83]. Interpretation of these associations should be made with caution as many studies have been conducted in highly selected patient populations. Thus, the generalizability of the reported estimates of malignancy risk and biomarker associations to broader populations of patients undergoing routine thyroid nodule evaluation is limited. In clinical practice, *RAS*-mutant thyroid tumors are often more indolent than *BRAF* V600E or telomerase reverse transcriptase (*TERT*)-mutated tumors, but the co-occurrence of *RAS* mutations with *TERT* promoter alterations is highly predictive of more aggressive tumors, recurrence, metastatic spread, and higher mortality [81,82,84].

#### 8.1.3. *RET*/*PTC* Fusions

Alterations in the rearranged during transfection (*RET*) gene, comprising both gene fusions and point mutations, are known oncogenic drivers in TCs, especially in PTC [85]. *RET* fusions are responsible for about 10–20% of PTC cases and show high incidence in radiation-induced thyroid tumors (approximately 58% in Chernobyl-related PTC), and a broader range reported across studies of 10.4–57.7% [85,86,87]. These rearrangements almost exclusively occur in PTC, where their incidence is significantly higher in pediatric and adolescent patients (29.8%) than in adults (8.7%), implicating childhood ionizing irradiation as an important cause [87]. *RET* fusions lead to a connection between the *RET* kinase domain and a promoter region of a partner gene, thus promoting kinase activity as a consequence of dimerization and ectopic expression in cells that normally do not express *RET* [85,87]. The most common fusions include coiled-coil domain-containing protein 6 (*CCDC6*)-*RET* and nuclear receptor coactivator 4 (*NCOA4*)-*RET* (*RET/PTC3*), which account for more than 90% of all detected *RET* fusions [85]. Bulanova Pekova et al. proved that the most common partner gene is *CCDC6* (59.3%), followed by *NCOA4* (22.1%), with 20 different types of *RET* rearrangements being discovered so far [87]. In clinical practice, PTCs associated with *RET* fusion show an aggressive behavior with increased incidence of lymph node metastases (75.2%), distant metastases (18.6%), extrathyroidal extension, and larger tumor size in *NCOA4-RET*(+) tumors [87]. Nonetheless, survival rates are relatively favorable despite the aggressive nature of PTCs related to *RET* fusion, with specific survival rates for the disease of 99%, 96%, and 95% at 2, 5, and 10 years, respectively [87]. Moreover, *RET* fusions play a critical role in TC formation induced by radiation and can be accurately identified using advanced molecular diagnostics that have become increasingly important for clinical practice [86]. This is confirmed by the recent introduction of highly selective *RET* inhibitors such as selpercatinib and pralsetinib [85,87].

### 8.2. Molecular Determinants of Tumor Behavior

The aggressiveness of thyroid tumors is determined not only by the presence of specific oncogenic drivers but also by the acquisition of additional molecular alterations during tumor evolution. While the early driver mutations, such as *BRAF* and *RAS*, create a favorable environment for tumor development via dysregulation of MAPK and PI3K/AKT pathways, the later genetic alterations, particularly *TERT* promoter and *TP53* mutations, seem to have a substantial influence on the phenotype and behavior of tumors [88]. TC development happens through a stepwise accumulation of molecular abnormalities that lead to the transition from DTC to poorly differentiated thyroid carcinoma (PDTC) and then to anaplastic thyroid carcinoma (ATC), the most genetically unstable type of thyroid tumors [82,88]. In turn, different genetic alterations influence the clinical outcome significantly. For instance, the co-occurrence of *TERT* promoter mutations along with *BRAF* V600E or *RAS* mutations increases the risk of recurrence, metastases, low disease-free and overall survival, and poorer prognosis [76,87,89]. Moreover, stromal proteins, like angiopoietin 2 (ANGPT2) and Delta-like canonical Notch ligand 4 (DLL4), may participate in tumor aggressiveness through promoting angiogenesis and vascular remodeling [82].

### 8.3. Epigenetic Modifications

Epigenetic modifications are changes in gene expression that do not affect the DNA sequence and are emerging as relevant contributors to TC formation. They are a putative mechanistic link between environmental exposures and the rise in TC incidence [90]. Increasing evidence shows the critical role of epigenetic dysregulation, including DNA and RNA methylation, histone modifications, and mechanisms related with micro ribonucleic acids (microRNAs) in the initiation, progression, and therapeutic response of thyroid tumors [90,91]. Aberrant DNA methylation may contribute to thyroid oncogenesis via hypermethylation of regulatory regions and subsequent silencing of tumor suppressor genes. Global DNA hypomethylation contributes to genomic instability and cancer progression [90]. Moreover, RNA methylation mechanisms, especially the N6-methyladenosine (m6A) modification, may control the stability and expression of genes involved in thyroid differentiation, such as Paired Box 8 (*PAX8*), further suggesting the involvement of epigenetic regulation in TC biology [90].

Environmental and metabolic factors seem to be major players in these epigenetic processes. Exposure to EDCs, heavy metals, persistent organic pollutants, per- and polyfluoroalkyl substances (PFASs), pesticides, and BPA has been associated with disruption of thyroid homeostasis and modified epigenetic regulation [92,93]. These compounds may interfere with the synthesis and transport of thyroid hormones, receptor signaling, and function of the hypothalamic–pituitary–thyroid (HPT) axis, resulting in thyroid hypertrophy, hormonal dysregulation, and possibly thyroid oncogenesis [91,93]. For example, BPA can mimic the endogenous hormones and compete with estrogens for receptor binding; on the other hand, phthalates and pesticides have been shown to alter DNA methylation patterns associated with endocrine disruption [91,93]. In addition, exposure to heavy metals such as cadmium, mercury, arsenic, lead, and manganese may result in endocrine signaling impairment and cause epigenetic abnormalities related to oxidative stress [92]. Environmental factors such as iodine deficiency and living in volcanic areas may also predispose people to TC through the combined effects of altered thyroid hormone signaling, heavy metal exposure, and toxicity related with radon [93]. Importantly, thyroid hormone itself can act as an environmental sensor that mediates epigenetic responses to external stimuli and hence integrates environmental signals with changes in gene expression and cellular phenotype [94].

Ionizing radiation has important epigenetic effects in addition to its direct mutagenic activity. Global DNA methylation patterns are altered in response to DNA damage induced by radiation, particularly double-strand breaks (DSBs). These changes may vary depending on the tissue type and the radiation exposure [95]. Such epigenetic dysregulation may interact with genomic rearrangements associated with radiation, such as *RET/PTC* fusions, which are typical molecular alterations in childhood and PTC related with radiation [91]. Of particular concern are epigenetic changes caused by environmental exposures and EDCs that may be maintained across generations, with potential transgenerational effects on thyroid health and cancer risk [92,93].

Epigenetic modifications, unlike irreversible genetic mutations, are potentially reversible and thus attractive therapeutic targets in TC [96]. Experimental studies have shown that epigenetic therapies can inhibit tumor growth and overcome treatment resistance. In PTC cells, SGI-1027 (the DNA methyltransferase inhibitor) decreased proliferation, migration, invasion, and glycolytic activity, while Panobinostat (a histone deacetylase inhibitor) induced apoptosis and cell cycle arrest in ATC models [96]. These findings suggest that targeting environmentally driven epigenetic pathways may represent a promising future strategy for TC prevention and treatment.

### 8.4. Clinical Implications of Molecular Alterations

The *BRAF* mutation status is a valuable molecular biomarker in TC, which provides important prognostic information and guides personalized therapeutic strategies. Besides its diagnostic utility, *BRAF* V600E is involved in improved risk stratification, influencing clinical decisions and patient management [76]. The prognostic significance of *BRAF* alterations is even more crucial when analyzed together with other molecular abnormalities. Mutations involving the *TERT* promoter, *TP53*, and the PI3K signaling pathway have been identified as strong predictors of poor clinical outcomes and reduced survival [89]. Among these, the co-occurrence of *BRAF* V600E with *TERT* promoter mutations defines a unique high-risk molecular subtype of PTC that demonstrates a synergistic activation of oncogenic and telomerase pathways, resulting in higher recurrence, distant metastasis, and disease-specific mortality [76,89]. The incorporation of *BRAF* and *TERT* genotyping into the AJCC/TNM staging system has been shown to significantly improve mortality risk stratification, especially in stage I–III disease patients, where the coexistence of both mutations can significantly alter risk classification [76]. Meanwhile, increasing evidence points to the critical role of epigenetic dysregulation in thyroid carcinogenesis, tumor progression, proliferation, and dedifferentiation, emphasizing the escalating importance of epigenetic mechanisms in TC pathogenesis [90,96].

The increasing knowledge of the genomics of TC has also changed diagnostic and therapeutic approaches. Commercially available molecular panels are increasingly used to augment the preoperative evaluation of cytologically indeterminate thyroid nodules, especially those classified as atypia of undetermined significance/follicular lesion of undetermined significance (AUS/FLUS), follicular neoplasm (FN), or suspicious for malignancy (SM) [82,83]. *BRAF* V600E detection is highly specific for malignancy and can substantially improve diagnostic accuracy in Bethesda III and IV nodules [76]. In addition, progress in precision oncology has resulted in the development of highly effective targeted therapies for advanced, recurrent, and radioiodine-refractory TCs [88]. Selective *RET* inhibitors, such as selpercatinib and pralsetinib, have shown remarkable clinical effectiveness and have been approved by the FDA for treatment-naïve and previously treated patients with *RET* alterations [85]. Similarly, TRK inhibitors (e.g., larotrectinib and entrectinib) are approved for solid tumors with *NTRK* gene fusions, irrespective of tissue origin [88]. Significant therapeutic progress has also been made with MAPK pathway inhibitors, with selective *BRAF* and *MEK* inhibitors, such as vemurafenib, dabrafenib, and selumetinib, showing clinical benefit in advanced TCs [76]. Dabrafenib and trametinib have become a standard of care for *BRAF* V600E-mutant ATC due to their significant survival benefit and have since been expanded to selected patients with papillary and poorly differentiated TCs [76].

## 9. Interaction of Contributing Factors

### 9.1. Multifactorial Model of TC Incidence

**Figure 1:**
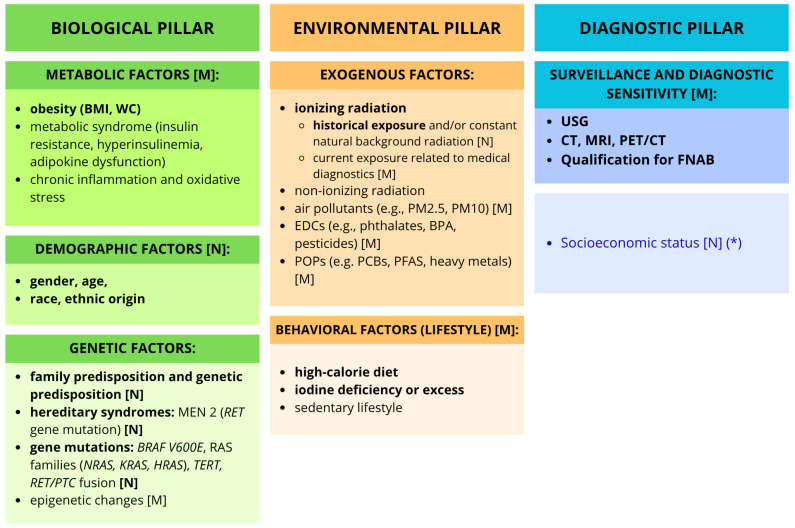
A three-pillar model of the multifactorial etiology of the increase in the number of TC cases. Explanations: Factors are classified as modifiable [M] or non-modifiable [N]. Family predisposition, although outside the main scope of this review, is shown as a constant biological background interacting with environmental exposures. Bold text denotes factors with an established epidemiological or pathophysiological association; regular font denotes hypothesis-generating factors that require further verification. The bold/regular convention applies only to the bulleted items within each category. Abbreviations used in the figure are defined in the manuscript-wide Abbreviations list. Socioeconomic status [N] (*) is a factor determining the diagnostic–biological balance rather than a direct risk factor (see Section 9.3).

The multifactorial model posits that the rise in TC incidence is the result of the dynamic interaction of multiple variables. It is now widely accepted that there is a synergistic relationship between factors in the biological, environmental, and diagnostic pillars (related to overdiagnosis) [2,33,97]. Figure 1 summarizes the proven and hypothetical risk factors that form the three pillars of the multifactorial etiology of the rising incidence of TC. A detailed discussion of the individual components is presented in Section 3, Section 4, Section 5, Section 6, Section 7 and Section 8.

### 9.2. Interactions Between Obesity and Environment

A key finding emerging from the literature review is that TC risk factors do not operate in isolation but interact in complex ways at multiple levels. The observed mechanism suggests the existence of a complex combined effect, which may manifest itself through both the cumulative and mutual, synergistic reinforcement of individual risk factors. Nevertheless, most available studies still analyze these variables independently of one another, and studies describing interactions among multiple risk factors are few [98,99].

One such study addressing this challenge is the cross-sectional study by Sada et al. (2025) [98], which directly analyzed the co-occurrence of multiple risk factors in patients with cytopathological changes in thyroid FNAB specimens. This study suggests the possibility of a significant metabolic–environmental correlation in the pathogenesis of DTC. The authors observed a linear relationship between waist circumference (WC) and the concentration of di(2-ethylhexyl) phthalate (DEHP) in the blood in a subgroup of obese patients (BMI > 30) in whom a FNAB revealed a high risk of malignancy in the nodules. They suggest that the anthropometric indicator WC may potentially serve as a more accurate predictor of this relationship than general BMI, presumably because it better reflects the mass of active visceral adipose tissue. The mechanism under analysis is based on the physicochemical properties of DEHP. Theoretically, as a highly lipophilic compound, DEHP exhibits a strong affinity for adipose tissue, suggesting that it may accumulate in adipocytes and then be gradually released into the bloodstream. This suggests that a larger volume of visceral fat may serve as a constant, systemic reservoir for this substance, leading to chronic exposure of the thyroid to its effects. This correlation supports the hypothesis of a possible interaction between exposure to EDCs and excess adipose tissue in the context of thyroid cancer risk. However, these conclusions are highly speculative and require verification in further prospective studies [98].

In the context of integrating environmental and biological factors, Sada’s (2025) [98] study aligns with the findings of Marotta et al. (2023) [99], serving as a methodological extension of their work. A key element of the model presented by Marotta’s team was the inclusion of metabolic syndrome in the statistical analysis, as after adjusting the results for its presence, the association between BPA exposure and TC in overweight and obese individuals (BMI ≥ 25 kg/m^2^) ceased to be statistically significant. On this basis, the researchers hypothesized that the carcinogenic effect of BPA on the thyroid does not necessarily result solely from direct thyrotoxicity. The likely mechanism is based on indirect, complex interactions of BPA with adipose tissue, which likely acts as a mediator between EDCs and the development of thyroid changes [99].

### 9.3. Diagnostics and True Disease Burden

In populations of highly developed countries with free-market healthcare systems, the diagnostic component is clearly dominant. Due to widespread access to diagnostic tools and screening programs, incidence statistics may be artificially inflated. The situation is different in countries with strong systemic regulation and restrictive screening guidelines, as well as in regions with lower socioeconomic status. In these populations, the diagnostic pillar gives way to the predominance of biological–environmental interactions. This also applies to areas with specific historical or environmental exposure characteristics, including past exposure to ionizing radiation following nuclear power plant accidents, regions with endemic iodine deficiency, areas of air pollution, or long-term exposure to EDCs. It is precisely these latter conditions that constitute a likely source of a real, biological increase in oncogenic potential [2,59,91,93].

A key methodological issue in research on the epidemiology of thyroid cancer is the problem of detection bias and the associated overdiagnosis. Detection bias is a statistical distortion resulting from excessive diagnostic vigilance in specific patient groups, which may mask or falsely exaggerate the actual influence of environmental factors on disease development [44]. In this context, a hypothesis has emerged that the association between obesity and thyroid cancer observed in the literature is spurious and stems solely from the fact that patients with a higher body mass index (BMI) seek medical care more frequently, which creates more opportunities for incidental detection of the tumor during ultrasound examinations. However, an analysis of scientific reports allows us to reject this theory and provides evidence that obesity itself constitutes a real, biological risk factor for the development of clinically significant and aggressive forms of thyroid cancer. To objectify the assessment and eliminate the influence of potential confounding factors, *BRAF* mutation status was used as a recognized tumor marker associated with higher malignant potential and poor clinical prognosis. The analysis showed that being overweight is associated with a significantly higher risk of developing *BRAF*-positive TC. The observed association between an elevated BMI and the presence of *BRAF* mutations seems to suggest that the issue is not merely due to a detection bias, but may have a genuine biological basis. If the observed epidemiological correlation were solely a result of intensified diagnostic surveillance in obese patients, the increased detection rate would apply equally to all lesion phenotypes—including clinically silent, indolent micronodules without *BRAF* mutations [44,100].

## 10. Future Research Directions

### 10.1. Reducing Overdiagnosis

Reducing overdiagnosis should remain a priority. Subsequent clinical recommendations should focus on limiting population-based, routine thyroid ultrasound screening in asymptomatic adults [97,101]. However, future studies must more precisely define the criteria for optimizing indications for fine-needle aspiration biopsy (FNAB). In this context, a research priority remains the rigorous validation of the 1 cm lesion size threshold in accordance with the guidelines of the American Thyroid Association (ATA) to standardize clinical practice across different healthcare systems [2,25,35].

Overdiagnosis predominates in high-income countries (HICs), while a genuine increase in incidence prevails in low- and middle-income countries (LMICs). This divergence requires international coordination of research and clinical efforts. It is essential to increase the diversity of clinical cohorts and strengthen the exchange of resources and technologies, such as telepathology and low-cost molecular diagnostics. This will enable the development of guidelines tailored to local systemic and genetic conditions [102].

### 10.2. Preventing Overtreatment

For low-risk tumors, clinical practice should focus on de-escalating surgical radicalism. Active surveillance (AS) is a key tool for this purpose; however, the widespread implementation of this strategy is limited by significant gaps in the current data [101,103]. AS protocols, validated primarily in Japanese cohorts, require further validation in Western populations through long-term observational studies evaluating oncological, psychological, and economic outcomes, including subpopulations of children, adolescents, and pregnant women [2,101,103,104,105,106]. Changing the clinical terminology to one that is less alarming, as has already been done in the case of non-invasive follicular thyroid neoplasm with papillary-like nuclear features, may further reduce surgical and psychological harm [34,103].

### 10.3. Research Prospects and Methodological Gaps in the Personalization of AS Strategies

Effective personalization of therapeutic strategies and safe patient selection for AS require the implementation of new diagnostic tools as well as the refinement of existing ones. There is a clear need for further research on new serological and molecular biomarkers [38,89]. Key areas of focus include, among others, the analysis of microRNA signatures as markers of aggressive microcarcinoma variants and the identification of signatures characteristic of specific environmental and biological factors. Another important step will be the implementation of risk stratification in AI-assisted ultrasound, as well as the development of clear guidelines regarding the predictive role of *BRAF* V600E and *TERT* promoter mutations in PTMC [101,103,105,107].

## 11. Conclusions

The global increase in thyroid cancer (TC) incidence is no longer viewed solely as a result of overdiagnosis. Although the detection of lesions with low malignant potential remains the primary cause in HICs, the concurrent increase in the number of advanced-stage diagnoses and mortality in certain populations indicates a genuine rise in incidence, rather than merely an artifact resulting from the scale of screening programs.

It is currently accepted that the development of TC has multifactorial origins, resulting from a complex network of interactions between biological and environmental factors and advances in diagnostics. However, it is essential to maintain a clear hierarchy between well-established and hypothetical factors. Well-documented risk factors include ionizing radiation, hereditary syndromes (e.g., MEN2), specific alterations (*BRAF* V600E, *RET/PTC* fusions), demographic variables, and abnormalities in iodine intake. Obesity is also a significant risk factor, although the observed association may be distorted to some extent by detection bias. In contrast, exposure to EDCs, air pollution, non-ionizing radiation, low physical activity, and metabolic syndrome remain hypothetical factors requiring direct molecular evidence—particularly regarding their epigenetic effects on thyrocytes—before they can be considered established determinants. Establishing this hierarchy not only organizes the current state of knowledge but also precisely identifies areas requiring urgent future analysis.

Clinically, this evolving understanding supports the reduction of unnecessary ultrasound examinations and biopsies in asymptomatic patients, while expanding active surveillance and molecular diagnostics for low-risk tumors. From a public health perspective, the priority should be to focus preventive measures on modifiable metabolic, environmental, and behavioral factors. Non-modifiable factors, on the other hand, should primarily be used for risk stratification in at-risk populations.

## Figures and Tables

**Table 1:** Endocrine-disrupting chemicals and thyroid effects.

Class of EDC	Thyroid-Related Effects
Phthalates	Endocrine disruption, interference with thyroid hormone regulation and cellular signaling
Bisphenol A (BPA)	Disruption of thyroid hormone synthesis, secretion and receptor-mediated signaling
Polychlorinated biphenyls (PCBs)	Disruption of thyroid hormone metabolism; possible association with thyroid cancer in occupational exposure studies

## Data Availability

No new data were created or analyzed in this study.

## Abbreviations

The following abbreviations are used in this manuscript:

8-OHdG	8-Hydroxy-2′-Deoxyguanosine
AdipoR	Adiponectin Receptor
AI	Artificial Intelligence
AKT	AKT Serine/Threonine Kinase (Protein Kinase B)
AMPK	Adenosine Monophosphate-activated Protein Kinase
ANGPT2	Angiopoietin 2
AS	Active Surveillance
ATA	American Thyroid Association
ATC	Anaplastic Thyroid Carcinoma
AUS	Atypia of Undetermined Significance
BMI	Body Mass Index
BPA	Bisphenol A
BRAF	B-Raf Proto-Oncogene
CCDC6	Coiled-Coil Domain Containing 6
CEUS	Contrast-Enhanced Ultrasound
CI	Confidence Interval
CT	Computed Tomography
DEHP	Di(2-Ethylhexyl) Phthalate
DLL4	Delta-Like Canonical Notch Ligand 4
DNA	Deoxyribonucleic Acid
DPP-4	Dipeptidyl Peptidase 4
DSBs	Double-Strand Breaks (in DNA)
DTC	Differentiated Thyroid Carcinoma
EDCs	Endocrine Disrupting Chemicals
EMT	Epithelial–Mesenchymal Transition
eNAMPT	extracellular Nicotinamide Phosphoribosyltransferase (visfatine)
EPA	Environmental Protection Agency
ERK	Extracellular Signal-Regulated Kinase
F-53B	Chlorinated Polyfluoroether Ether Sulfonate
FLUS	Follicular Lesion of Undetermined Significance
FN	Follicular Neoplasm
FNAB	Fine-Needle Aspiration Biopsy
FTC	Follicular Thyroid Carcinoma
FVPTC	Follicular Variant Papillary Thyroid Carcinoma
GenX	Ammonium salt of hexafluoropropylene oxide dimer acid
GIP	Gastric Inhibitory Polypeptide
GLP-1	Glucagon-Like Peptide 1
Gy	Gray (the SI unit of absorbed ionizing radiation dose)
HICs	High-Income Countries
HIF-1	Hypoxia-Inducible Factor 1
HMGA1	High Mobility Group AT-Hook 1
HPT axis	Hypothalamic-Pituitary-Thyroid axis
HRAS	Harvey Rat Sarcoma Viral Oncogene Homolog
HRUS	High-Resolution Ultrasound
hTERT	Human Telomerase Reverse Transcriptase
IARC	International Agency for Research on Cancer
IFN	Interferon
IGF	Insulin-like Growth Factor
IGFBP	Insulin-like Growth Factor-Binding Protein
IKK	IκB Kinase
IL	Interleukin
INSR	Insulin Receptor
IR	Insulin Resistance
JAK2	Janus Kinase 2
JNK	c-Jun N-terminal Kinase
KRAS	Kirsten Rat Sarcoma Viral Oncogene Homolog
LMICs	Low- and Middle-Income Countries
m6A	N6-methyladenosine
MAPK	Mitogen-Activated Protein Kinase
MEK	Mitogen-Activated Protein Kinase Kinase
MEN2	Multiple Endocrine Neoplasia type 2
MeSH	Medical Subject Headings
microRNA	Micro Ribonucleic Acid
MRI	Magnetic Resonance Imaging
mRNA	Messenger Ribonucleic Acid
MTC	Medullary Thyroid Carcinoma
mTOR	Mammalian Target of Rapamycin
NAD+	Nicotinamide Adenine Dinucleotide (oxidized form)
NCOA4	Nuclear Receptor Coactivator 4
NF-κB	Nuclear Factor kappa-light-chain-enhancer of activated B cells
NIFTPs	Non-invasive Follicular Thyroid Neoplasm with Papillary-like Nuclear Features
NIR	Non-ionizing Radiation
NIS	Sodium/Iodide Symporter
NRAS	Neuroblastoma RAS Viral Oncogene Homolog
Ob-R	Leptin Receptor (Obese Receptor)
OR	Odds Ratio
PAX8	Paired Box 8
PBEF	Pre-B-cell Colony-Enhancing Factor (visfatine)
PCBs	Polychlorinated Biphenyls
PDTC	Poorly Differentiated Thyroid Carcinoma
PET	Positron Emission Tomography
PFASs	Per- and Polyfluoroalkyl Substances
PI3K	Phosphoinositide 3-Kinase
PIK3R1	Phosphoinositide-3-Kinase Regulatory Subunit 1
POPs	Persistent Organic Pollutants
PM	Particulate Matter
PPARγ	Peroxisome Proliferator-Activated Receptor Gamma
PTC	Papillary Thyroid Carcinoma
PTMC	Papillary Thyroid Microcarcinoma
RAF	Serine/Threonine-Protein Kinase
RAI	Radioactive Iodine
RARRES2	Retinoic Acid Receptor Responder 2
RET	Rearranged during Transfection
RETN	Resistin
RNA	Ribonucleic Acid
ROS	Reactive Oxygen Species
RXRA	Retinoid X Receptor Alpha
SEER	Surveillance, Epidemiology, and End Results Program
SIRT3	Sirtuin 3
SM	Suspicious for Malignancy
STAT3	Signal Transducer and Activator of Transcription 3
T2DM	Type 2 Diabetes Mellitus
TC	Thyroid Cancer
TERT	Telomerase Reverse Transcriptase
TLR	Toll-Like Receptor
TNF-α	Tumor Necrosis Factor Alpha
TP53	Tumor Protein p53
TR	Thyroid Hormone Receptor
US	Ultrasound
VEGF	Vascular Endothelial Growth Factor
WC	Waist Circumference
X-ray	X-radiation
